# Circulating microRNA124-3p, microRNA9-3p and microRNA196b-5p may be potential signatures for differential diagnosis of thyroid nodules

**DOI:** 10.18632/oncotarget.12389

**Published:** 2016-10-01

**Authors:** Sui Yu, Xiaoling Liu, Yifei Zhang, Jing Li, Shulin Chen, Haitao Zheng, Ruizhen Reng, Chenglin Zhang, Jian Chen, Li Chen

**Affiliations:** ^1^ Qilu Hospital, Shandong University, Jinan, Shandong, China; ^2^ The Key Laboratory of Cardiovascular Remodeling and Function Research, Chinese Ministry of Education and Chinese Ministry of Health, The State and Shandong Province Joint Key Laboratory of Translational Cardiovascular Medicine, Qilu Hospital of Shandong University, Jinan, Shandong, China; ^3^ The Affiliated Yantai Yuhuangding Hospital of Qingdao University, Yantai, Shandong, China

**Keywords:** papillary thyroid carcinoma, circulating microRNAs, miR-124-3p, miR-9-3p, miR-196b-5p

## Abstract

It is important to develop an effective auxiliary approach to distinguish papillary thyroid carcinoma (PTC) from benign nodules because a considerable proportion cannot be identified by fine-needle aspiration cytology at present, resulting in unnecessary thyroidectomy. Circulating miRNAs are potential biomarkers for differential diagnosis of tumors. We aimed to investigate the dysregulation of circulating miRNAs in PTC and evaluate the diagnostic value for differentiation of PTC from benign nodules. We first assessed the expression of miRNAs in patients with PTC, patients with benign nodules and healthy controls using a miRCURY LNA Array (*n* = 3 for each group). Expression of circulating miR-124-3p, miR-9-3p and miR-5691 was significantly up-regulated, while miR-4701 and miR-196b-5p were down-regulated in PTC patients. The dysregulation of miR-124-3p, miR-9-3p, miR-4701 and miR-196b-5p was further validated by qRT-PCR in fifty participants from each group. The expression of circulating miR-124-3p and miR-9-3p was significantly up-regulated in PTC patients. Both miR-124-3p and miR-9-3p could distinguish PTC from benign nodules with high sensitivity and specificity. There were no significant differences in the expression of circulating miR-4701 and miR-196b-5p between PTC patients and healthy controls. Nevertheless, patients with benign nodules showed a higher level of miR-196b-5p compared with that of PTC patients and healthy controls. ROC analysis indicated that miR-196b-5p had a good diagnostic value for differentiation of benign nodules from PTC. Our study suggested that miR-124-3p, miR-9-3p and miR-196b-5p may be potential signatures for differential diagnosis of thyroid nodules in eastern coastal areas of China.

## INTRODUCTION

The incidence of thyroid nodules has been significantly increasing due to intensive screening by neck ultrasounds in recent decades [1]. Most thyroid nodules are benign, and only 7%–15% cases are malignant and require surgical intervention [2]. Papillary thyroid carcinoma (PTC) is the most common type of thyroid cancer (approximately 80%) [2]. Thus, it is very important to distinguish malignant nodules from benign ones to avoid excessive treatments. Fine-needle aspiration cytology (FNAC) is currently the best approach for the differential diagnosis of thyroid nodules. However, 10%– 40% of thyroid nodules cannot be distinguished by FNAC, which may be due to inadequate or inaccurate sampling or the difficulty in distinguishing follicular lesions [2]. This would result in a relatively blind treatment and possibly an unnecessary thyroidectomy. Therefore, improving the differential accuracy of thyroid nodules is a vital issue that urgently needs to be addressed.

MicroRNAs (miRNAs) are small, endogenous, noncoding RNA molecules that post-transcriptionally regulate gene expression via inhibition of the target mRNAs. MiRNAs can participate in most cellular processes, including cell growth, differentiation, apoptosis, and adhesion. Accumulating studies have shown that miRNA expression is dysregulated in many types of malignant tumors, including thyroid cancers [3]. Furthermore, miRNAs exhibit superior stability and maintain their expression profiles in formalin-fixed tissue samples [4]. Taken together, these findings indicate that miRNAs are potential biomarkers for differential diagnosis. Tetzlaff et al. found 13 up-regulated and 26 down-regulated miRNAs in PTC compared with multinodular goiters in formalin-fixed tissues [5]. Other studies demonstrated that compared with the tissue samples from benign nodules, PTC samples showed up-regulation of miRNA-146b, miRNA-221, miRNA-222, miRNA-181b and miRNA-155, in some cases by more than 10-fold [6–8]. However, although tissue miRNA profiles may be useful for the differential diagnosis of thyroid nodules, acquiring tissue samples is an invasive procedure, and the accuracy partly depends on accurate sampling, the same limiting factor as FNAC.

In contrast, circulating miRNAs released from disease cells can avoid degradation and are highly stable; thus, they can be detected in plasma or serum [9]. This suggests the potential of circulating miRNAs to act as biomarkers for differential diagnosis of tumors. In recent years, several studies have shown the diagnostic value and advantages of circulating miRNAs as minimally invasive biomarkers in many types of cancers, such as lung, gastrointestinal and breast cancers [10–12]. A 2012 study investigated the circulating miRNA expression in PTC patients for the first time [13]. They found serum let- 7e, miRNA-151-5p, and miRNA-222 were significantly up-regulated in PTC patients compared with those of benign cases and healthy controls in southern China. This supported the hypothesis that circulating miRNAs are an easy, minimally invasive, and effective auxiliary biomarker for the preoperative diagnosis of thyroid nodules. However, to date, there are few of studies on circulating miRNAs of PTC patients, and the dysregulated serum miRNAs in PTC showed poor consistency. Cantara et al. found that miRNA-95 was down-regulated, and miRNA-190 was up-regulated in a Caucasian population [14]. Li et al. reported that miRNA-25-3p and miRNA-451a were up-regulated in PTC patients and decreased after tumor excision in northern China [15]. More studies are needed to elucidate the dysregulation of circulating miRNAs and provide sufficient evidence to develop them as an easy, non-invasive and effective differential tool for the preoperative diagnosis of thyroid nodules. The causes of the circulating miRNA inconsistency in different studies on PTC are not known. Regional differences may be one possible explanation. Epidemiological evidence indicates that excess iodine is probably one of the external environmental factors causing thyroid cancer prevalence. The eastern coastal area of China is iodine-rich and shows a higher incidence of thyroid cancer. To date, there have no studies on the alteration of circulating miRNAs in eastern coastal areas of China.

In the present study, we aimed to investigate the dysregulation of circulating miRNAs and evaluate the potential value of differential diagnosis in PTC patients from eastern coastal areas of China.

## RESULTS

### Clinical characteristics

A total of 150 participants were enrolled in the present study, including 50 patients with primary PTC, 50 with benign thyroid nodules and 50 age- and gender-matched healthy controls. The pathological types of PTC included 46 with the classical variant, 3 with the follicular variant and 1 with the sclerosis variant of PTC. The clinical characteristics for all participants were recorded. As shown in Table 1, the three groups did not differ in age, sex, obesity, heart rates, thyroid functions, and other basic laboratory parameters, including liver function, kidney function and fasting plasma glucose.

**Table 1:** Clinical characteristics of the study population in three groups

Characteristic	Control (*n* = 50)	Benign (*n* = 50)	PTC (*n* = 50)
**Age** (years)	40.44 ± 1.57	44.68 ± 2.01	40.98 ± 1.55
**Sex** (female, %)	39 (78.0%)	40 (80.0%)	40 (80.0%)
**BMI** (kg/m^2^)	22.77 ± 0.41	23.00 ± 0.44	23.53 ± 0.37
**Heart rates**(bpm)	76.48 ± 1.20	79.02 ± 1.23	76.22 ± 1.02
**ALT** (U/L)	19.20 ± 1.10	17.38 ± 0.99	21.14 ± 2.18
**Creatinine**(μmol/L)	57.96 ± 1.72	53.04 ± 1.77	57.34 ± 2.36
**FPG**(mmol/L)	4.90 ± 0.08	4.80 ± 0.13	4.90 ± 0.12
**TSH** (mIU/L)	2.00 ± 0.11	2.24 ± 0.16	2.47 ± 0.20
**FT4** (pmol/L)	16.58 ± 0.27	16.59 ± 0.26	15.89 ± 0.34
**FT3** (pmol/L)	4.83 ± 0.07	5.05 ± 0.09	4.91 ± 0.07

Data are shown as the mean ± SEM or number [%] of participants. Bpm, beats per minute; BMI, body mass index; ALT, alanine aminotransferase; FPG, fasting plasma glucose; TSH, thyroid-stimulating hormone; FT3, free triiodothyronine; FT4, free tetraiodothyronine. There were no significant differences in these clinical characteristics among the three groups.

### Dysregulated circulating miRNAs screened by a miRCURY LNA Array in PTC patients

We measured the expression of circulating miRNAs in 3 participants from each group using a miRCURY LNA Array. A total of 2085 miRNAs were analyzed, and 957 were detected in all participants, while 676 showed no expression in the three groups. We performed further analysis with the 957 stably expressed miRNAs in plasma.

**Figure 1:**
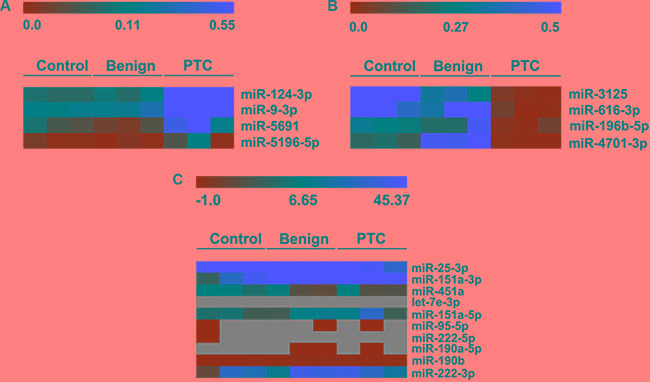
Hierarchical clustering and heat map analysis of miRNA array profiling (**A**) The heat map of miRNAs significantly up-regulated in PTC patients. The change in miRNA expression in PTC compared to benign nodules is larger than 10-fold. (**B**) Heat map of miRNAs down-regulated in PTC patients. The change in miRNA expression in PTC compared to benign nodules is less than 0.2-fold. (**C**) Heat map of circulating miRNAs reported in other studies. Each row represents a detected miRNA. The scale color from green (low expression) to red (high expression) indicates the expression levels of each miRNA. Gray represents loss of expression.

Hierarchical clustering and heat map analysis of the potential dysregulated miRNAs are shown in Figure 1A and 1B. We searched for potential miRNAs to distinguish PTC patients from those with benign nodules and healthy volunteers based on the following criteria: 1) for up-regulated plasma miRNAs in PTC patients, the expression of targeted miRNA in PTC patients is increased by more than 10-fold compared to that of patients with benign nodules (PTC/Benign > 10), while the expression in patients with benign nodule is more than 0.5– and less than 2-fold of that in healthy controls (0.5 < Benign/Control < 2); 2) for down-regulated plasma miRNAs, the expression of targeted miRNAs in PTC patients is less than 0.2-fold compared to that of patients with benign nodules (PTC/Benign < 0.2), while the expression in patients with benign nodule is more than 0.5-fold of that in healthy controls (Benign/Control > 0.5). Based on these criteria, we identified 3 up-regulated miRNAs (miR- 124- 3p, miR- 9-3p, miR-5691) and 2 down-regulated miRNAs (miR-4701, miR-196b-5p) that distinguished the PTC patients from the patients with benign nodules and healthy controls (Table 2).

**Table 2:** Dysregulated circulating miRNAs in PTC patients screened by miRCURY LNA array

miRNAs	Normalized expression (mean ± SEM)			Fold change (Mean)		
	Control (*n* = 3)	Benign (*n* = 3)	PTC (*n* = 3)	Benign/Control	PTC/Control	PTC/Benign
**Up-regulated**						
miR-124-3p	0.10 ± 0.01	0.08 ± 0.00	23.01 ± 6.46/*	1.21	273.56	225.01
miR-9-3p	0.19 ± 0.04	0.14 ± 0.01	14.30 ± 5.05*	1.37	101.59	73.84
miR-5691	0.04 ± 0.01	0.06 ± 0.02	0.47 ± 0.19*	0.76	8.29	10.97
**Down-regulated**						
miR-4701	0.50 ± 0.03	0.19 ± 0.01*	0.01 ± 0.00*	2.65	0.05	0.02
miR-196b-5p	0.30 ± 0.09	0.28 ± 0.01	0.03 ± 0.02*	1.08	0.12	0.11

* *P* < 0.05, vs control.

We also investigated the expression of circulating miRNAs reported in the previous studies, including let- 7e (let-7e-3p), miRNA-151-5p (miR-151a-3p, miR-151a- 5p), miRNA-222 (miR-222-5p, miR-222-3p), miRNA-95 (miR-95-5p), miRNA-190 (miR-190b, miR-190a-5p), miRNA-25-3p and miRNA-451a, in the three groups in the present study (Figure 1C). We found that there was no detectable expression of circulating let- 7e- 3p, miR-222- 5p, and miR-95-5p in the three groups. The circulating level of miR-190a-5p was very low in healthy controls but undetectable in both patients with benign nodules or PTC patients. MiR-190b showed a very low expression, while miR-151a-3p and miR-151a-5p, miR-222-3p, miR-25-3p, miR-451a had stable and high expression; however, there were no significant differences among the three groups (Table 3).

**Table 3:** Expression of circulating miRNAs reported in previous studies in PTC patients

miRNAs	Normalized expression (mean ± SEM)			Fold change (Mean)		
	Control (*n* = 3)	Benign (*n* = 3)	PTC (*n* = 3)	Benign/Control	PTC/Control	PTC/Benign
let-7e-3p	N/A	N/A	N/A	N/A	N/A	N/A
miR-151a-5p	14.94 ± 3.95	5.2 ± 0.09	8.56 ± 1.25	2.87	1.65	0.57
miR-151a-3p	46.56 ± 1.72	33.24 ± 1.07	24.07 ± 11.16	1.40	0.72	0.52
miR-222-5p	N/A	N/A	N/A	N/A	N/A	N/A
miR-222-3p	26.46 ± 1.94	15.68 ± 2.01	17.91 ± 7.66	1.69	1.14	0.68
miR-95-5p	N/A	N/A	N/A	N/A	N/A	N/A
miR-190b	0.33 ± 0.04	0.28 ± 0.00	0.14 ± 0.09	1.19	0.50	0.42
miR-190a-5p	0.017 ± 0.01	N/A	N/A	N/A	N/A	N/A
miR-25-3p	40.45 ± 3.91	37.84 ± 0.85	45.32 ± 3.05	1.07	1.20	1.12
miR-451a	3.76 ± 0.17	6.62 ± 1.33	8.87 ± 0.10	0.57	1.34	2.36

There were no significant differences in the expression of the above miRNAs among the three groups. N/A, not applicable.

### Dysregulated circulating miRNAs in PTC patients validated by qRT-PCR

**Figure 2:**
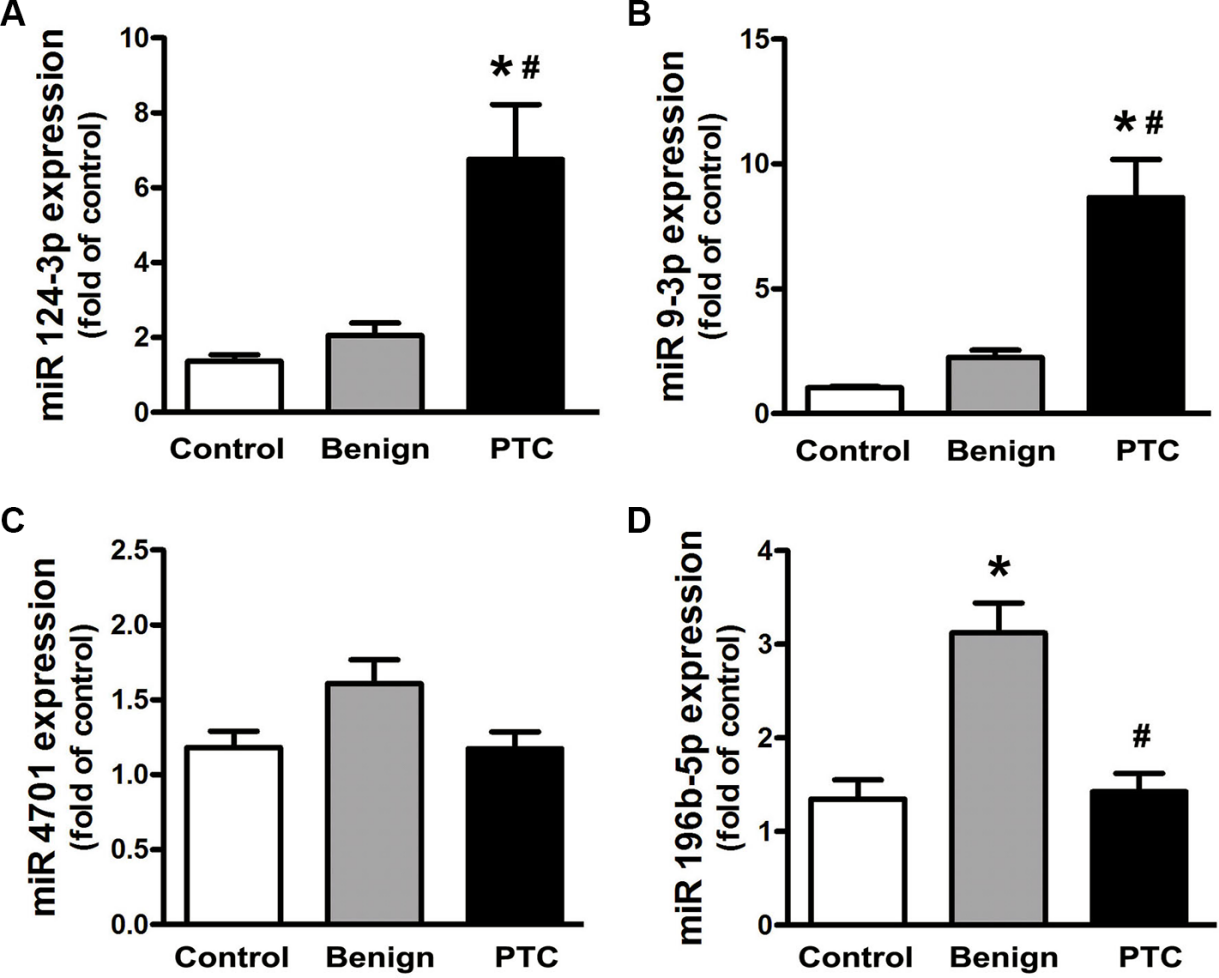
Expression of miR-124-3p, miR-9-3p, miR-4701 and miR-196b-5p measured by qRT-PCR Data are expressed as the mean ± SEM. *N* = 50 for each group. *P < 0.05, vs. control. ^#^*P* < 0.05, vs. benign.

As shown in Table 2, among three up-regulated plasma miRNAs, miR-5691 exhibited very low plasma levels but was up-regulated in PTC patients, which would result in poor sensitivity and specificity. In contrast, the expression of miR-124-3p and miR-9-3p was much higher in PTC patients but very low in patients with benign nodules and healthy controls. Therefore, we selected miR-124-3p and miR-9-3p as the potential up-regulated miRNA signatures for further qRT-PCR validation. In other words, because both miR-4701 and miR-196b-5p showed very low plasma expression in the three groups, we had to validate both down-regulated miRNAs. Thus, we measured the expression of circulating miR-124-3p, miR-9-3p, miR-4701 and miR-196b-5p using qRT-PCR in patients with PTC or benign nodules and healthy controls. We found that the plasma levels of miR-124-3p and miR- 9-3p in PTC patients were significantly higher than those in patients with benign nodules and healthy controls, while there were no significant differences between patients with benign nodules and healthy controls (Figure 2A, 2B). In contrast, the expression of circulating miR-4701 and miR-196b-5p showed poor consistency with the results from the miRCURY LNA Array screening. There were no significant differences in the expression of circulating miR-4701 among the three groups (*P* > 0.05, Figure 2C). For miR-196b-5p, patients with benign nodules had a higher plasma level than that of healthy controls and PTC patients (*P* < 0.05); however, there was no significant difference between the healthy controls and PTC patients (*P* > 0.05, Figure 2D).

### Expression of miR-124-3p, miR-9-3p and miR-196b-5p in thyroid tissues

**Figure 3:**
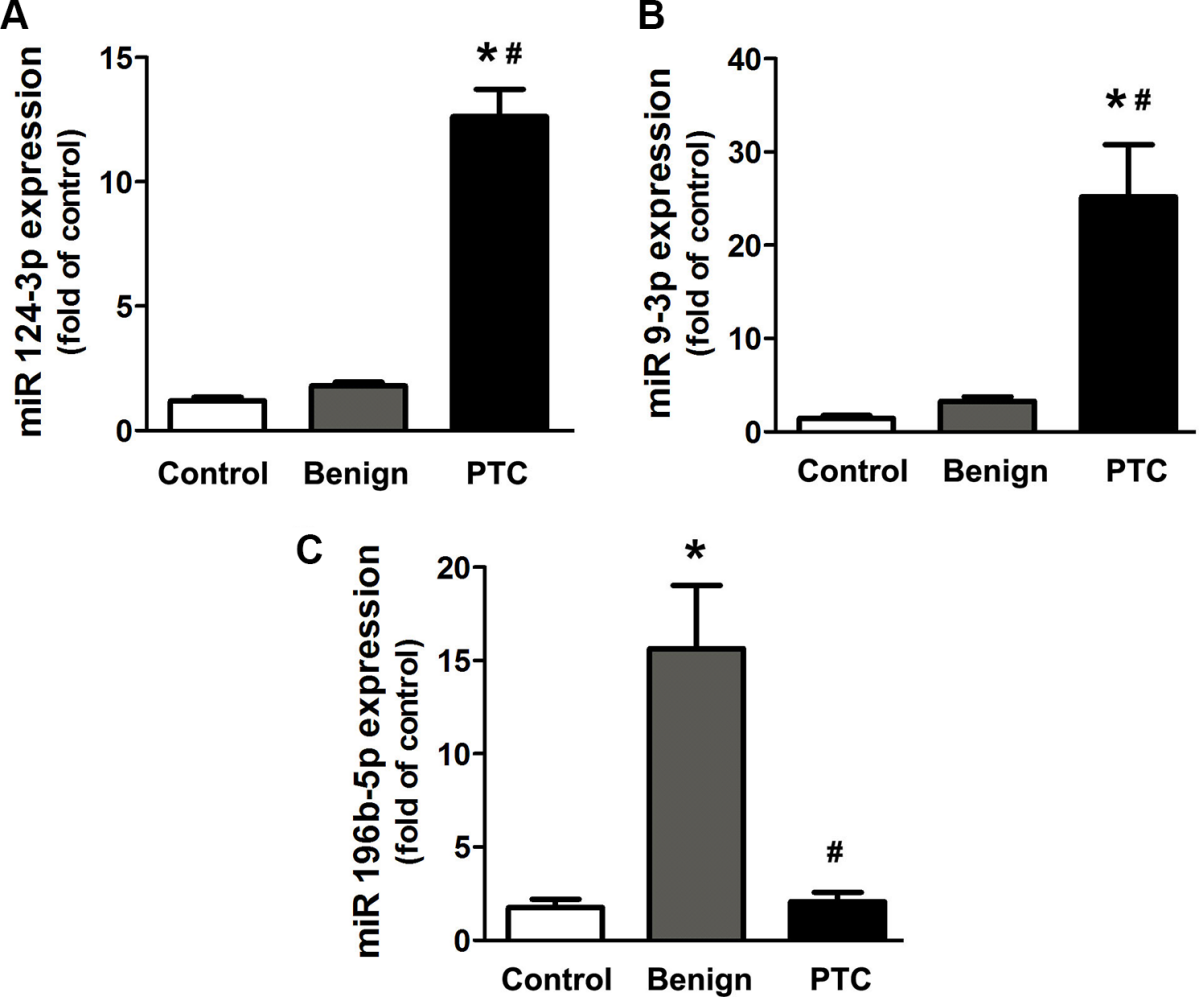
Expression of miR-124-3p, miR-9-3p and miR-196b-5p in thyroid tissues Data are expressed as the mean ± SEM. *N* = 27 for each group. *P < 0.05, vs. control. ^#^*P* < 0.05, vs. benign.

To further investigate the expression of these dysregulated circulating miRNAs in thyroid nodules, we performed qRT-PCR with thyroid tissues from patients who received a thyroidectomy. We found that the expression of miR-124-3p and miR-9-3p was markedly increased in PTC tissues compared with tissues with benign nodules and the normal thyroid tissues (Figure 3A, 3B). There were no significant differences in the expression of miR-196b-5p between PTC and normal thyroid tissues. However, tissues with benign nodules showed a markedly increased expression of miR-196b-5p compared to that of both the PTC and the normal samples (Figure 3C). These results showed a consistent trend of dysregulated miRNAs in tissues and the plasma.

### Expression of miR-124-3p and miR-9-3p after thyroidectomy

**Figure 4:**
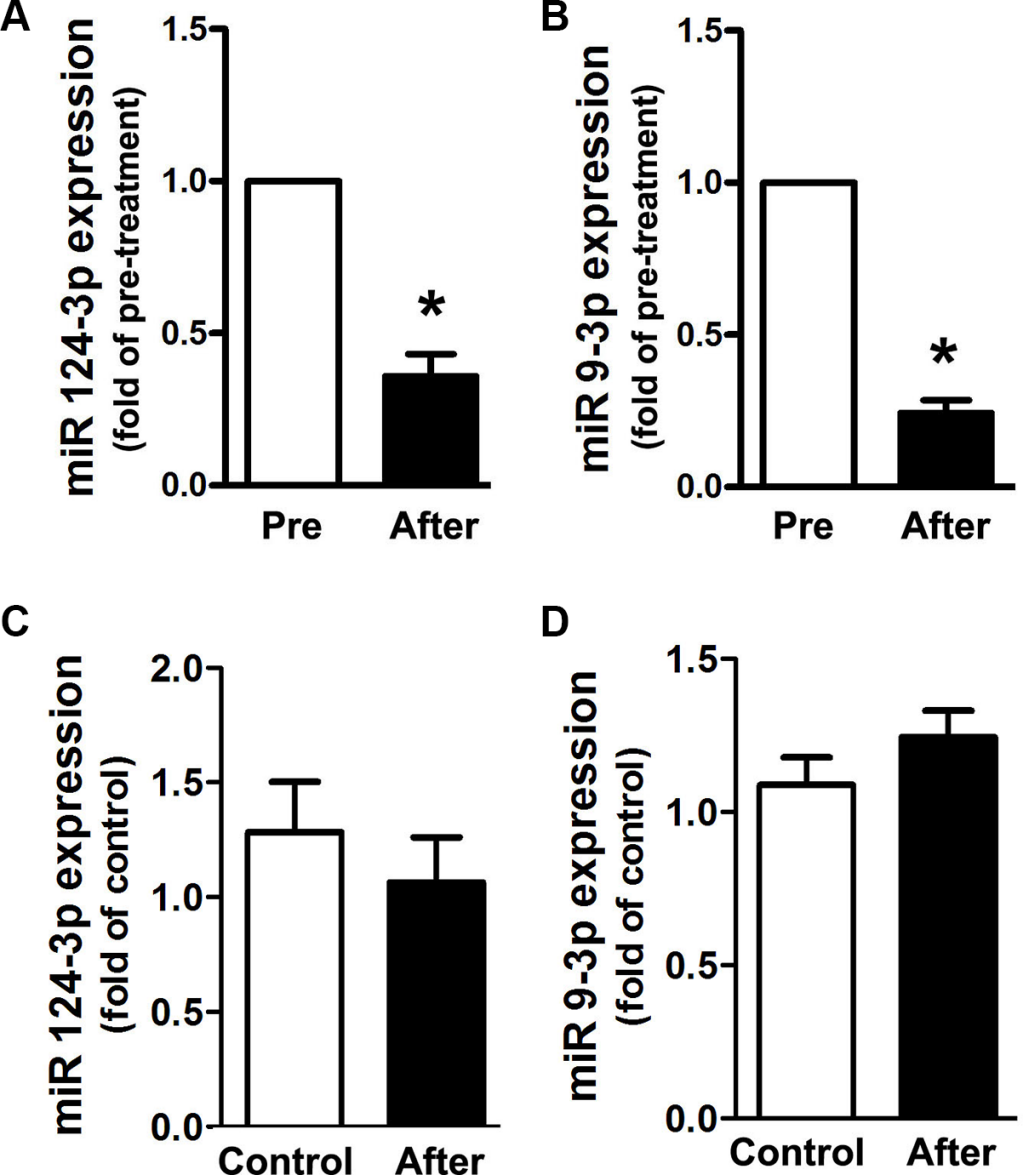
Expression alteration of circulating miR-124-3p and miR-9-3p in PTC patients 4 weeks after thyroidectomy (A, B) Alteration in the expression of circulating miR-124-3p and miR-9-3p in PTC patients 4 weeks after thyroidectomy (after) compared with the level of pre-treatment (pre). *P < 0.05, vs. pre-treatment. (**C**, **D**). Expression of circulating miR-124-3p and miR-9-3p in PTC patients 4 weeks after thyroidectomy compared with those in healthy volunteers (control).

To elucidate the alteration of circulating miR- 124- 3p and miR-9-3p expression in PTC patients after thyroidectomy, we followed up the patients and collected plasma for qRT-PCR analysis 4 weeks after the surgery. Compared to pre-treatment samples, the expression of both miR-124-3p and miR-9-3p in plasma was significantly decreased to 36% and 24%, respectively, 4 weeks after thyroidectomy (Figure 4A and 4B). Additionally, the expression of these two circulating miRNAs in PTC patients after thyroidectomy showed no significant difference compared to that in healthy volunteers (Figure 4C and 4D).

### Diagnostic value of circulating miRNAs for PTC

**Figure 5:**
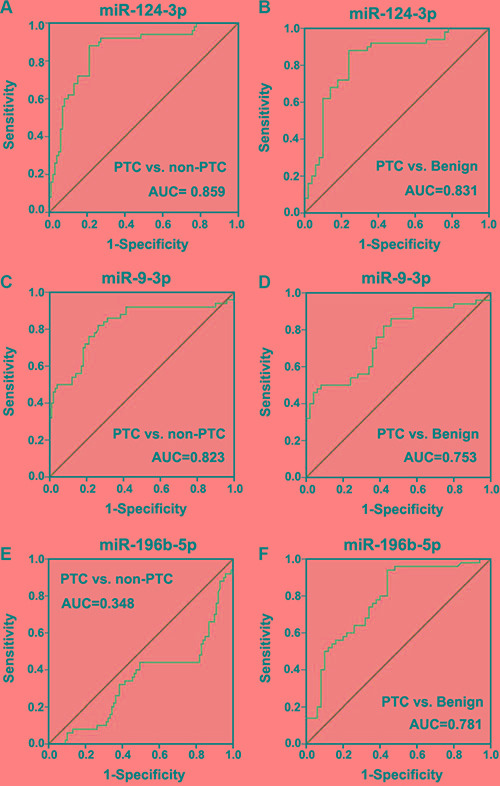
Receiver operating characteristic (ROC) curve of miR-124-3p, miR-9-3p and miR-196b-5p for the diagnostic value of differentiation of PTC patients from patients with benign nodules or healthy controls

To evaluate the diagnostic value of circulating miR-124-3p, miR-9-3p and miR-196b-5p, ROC analysis was performed. In the comparison of PTC and non-PTC groups, miR-124-3p showed an area under the ROC curve (AUC) of 0.859 [95% confidence interval (CI) = 0.794– 0.923], with 88% sensitivity and 78.8% specificity at the cutoff value of 2.04 (Figure 5A). Further comparison between PTC patients and benign nodules showed that miR-124-3p had an AUC of 0.831 (95% CI = 0.747–0.915), with 88% sensitivity and 76% specificity at the cutoff value of 2.04 (Figure 5B).

Circulating miR-9-3p had an AUC of 0.823 (95% CI = 0.743–0.902), with 80% sensitivity and 73.7% specificity at the cutoff value of 1.70 in the comparison between PTC and non-PTC groups (Figure 5C). The comparison between PTC and benign groups showed that miR-9-3p had an AUC of 0.753 (95% CI = 0.657–0.849), with 70% sensitivity and 64% specificity at the cutoff value of 2.09 (Figure 5D).

ROC analysis indicated that miR-196b-5p was unable to distinguish PTC patients from non-PTC subjects (Figure 5E). Nevertheless, miR-196b-5p had an AUC of 0.781 (95% CI = 0.690–0.872), with 74% sensitivity and 66% specificity at the cutoff value of 1.545 in the comparison of patients with benign nodules and PTC patients, which indicated the potential of miR-196b-5p to distinguish benign nodules from PTC in patients with thyroid nodules (Figure 5F).

### Correlation of circulating miRNA expression and clinicopathological features in PTC

To determine whether the dysregulation of miRNA expression in PTC patients was associated with clinicopathological features, we further analyzed the expression of miR-124-3p, miR-9-3p and miR-196b-5p in different subgroups divided by age, gender, tumor-related pathological characteristics or BRAF gene mutation, which is the most important proto-oncogene in PTC. As shown in Table 4, the expression of miR-124-3p was up-regulated in younger patients (< 45 years old) or patients with a tumor size larger than 2 cm. The expression of miR-9-3p also exhibited a significant up-regulation in younger patients. The expression of miR-196b-5p was not associated with different clinicopathological characteristics.

**Table 4:** Expression of miR-124-3p, miR-9-3p, and miR-196b-5p in subgroups divided by clinicopathological features in PTC patients

Characteristic	Number of patients	miR-124-3p	miR-9-3p	miR-196b-5p
**Gender**				
Female	40	6.39 ± 1.62	8.82 ± 1.86	1.32 ± 0.91
Male	10	8.20 ± 3.57	8.01 ± 1.70	1.79 ± 0.55
**Age**				
< 45 years old	29	8.99 ± 2.44	11.38 ± 2.41	1.35 ± 0.34
≥ 45 years old	21	3.66 ± 0.44*	4.90 ± 1.01*	1.48 ± 0.24
**Tumor size**				
≤ 1 cm	19	3.67 ± 0.52	9.10 ± 3.18	1.24 ± 0.29
1–2 cm	17	3.89 ± 0.49	8.42 ± 1.81	1.59 ± 0.42
≥ 2 cm	14	14.42 ± 4.66#†	8.34 ± 2.60	1.46 ± 0.31
**Lymph node metastasis**				
No	26	7.33 ± 2.42	9.96 ± 2.47	1.35 ± 0.23
Yes	24	6.13 ± 1.59	7.25 ± 1.71	1.51 ± 0.34
**Extrathyroidal extension**				
No	24	4.79 ± 0.58	11.01 ± 2.55	1.68 ± 0.27
Yes	26	9.56 ± 3.53	6.49 ± 1.68	1.14 ± 0.29
**Tumor multifocality**				
No	36	4.88 ± 0.54	7.59 ± 1.37	1.44 ± 0.23
Yes	14	11.58 ± 4.93	11.42 ± 4.17	1.39 ± 0.38
**Tumor bilaterality**				
No	41	4.79 ± 0.49	8.63 ± 1.66	1.50 ± 0.24
Yes	9	15.68 ± 7.44	8.78 ± 4.02	1.10 ± 0.22
**TNM stage**				
I/II	38	6.14 ± 1.69	8.29 ± 1.88	1.26 ± 0.20
III/IV	12	8.69 ± 2.97	9.83 ± 2.28	1.96 ± 0.53
**BRAF mutation**				
No	14	5.08 ± 1.00	10.50 ± 3.24	1.27 ± 0.21
Yes	36	7.41 ± 1.99	7.94 ± 1.71	1.48 ± 0.26

Data are shown as the mean ± SEM or number of participants. TNM, tumor node metastases.

* *P* < 0.05, vs. patients younger than 45 years old

# *P* < 0.05, vs. patients with tumor size ≤ 1 cm

† *P* < 0.05, vs. patients with tumor size 1–2 cm.

## DISCUSSION

In the present study, we enrolled patients with thyroid nodules to analyze the dysregulation of circulating miRNAs in patients with PTC in an eastern coastal area of China and investigate the diagnostic value to differentiate PTC from benign nodules. We found that the expression of plasma miR-124-3p and miR-9-3p was significantly up-regulated compared with that of patients with benign nodules and healthy controls. The expression of these circulating miRNAs exhibited a similar dysregulation in the thyroid tissues. The expression of circulating miR-124-3p and miR-9-3p in PTC patients was decreased significantly after thyroidectomy. Both miR-124-3p and miR-9-3p exhibited a strong diagnostic ability to distinguish PTC from benign nodules with high sensitivity and specificity. miR-4701 and miR-196b-5p were found to be down-regulated in PTC patients by miRCURY LNA Array screening; however, there was inconsistency in the further validation by qRT-PCR. This might be due to the very low plasma levels, resulting in poor sensitivity and specificity. Nevertheless, both miRCURY LNA Array screening and qRT-PCR results confirmed that the expression of miR-196b-5p was up-regulated in patients with benign nodules compared to that in healthy controls and PTC patients. ROC analysis revealed that miR- 196b- 5p had the potential to differentiate patients with benign nodules from PTC patients with a high sensitivity and specificity. Our study suggested that miR-124-3p, miR-9-3p and miR-196b-5p might be a potential signature for the differential diagnosis of thyroid nodules in eastern coastal areas of China.

MiR-124 was first found to be involved in neuronal differentiation and stem cell regulation [16, 17]. Many studies have demonstrated that miR-124 is a critical regulator of tumor development and progression. The expression of miR-124 was down-regulated in various types of cancer, such as gastric cancer, colorectal cancer, glioblastoma, and breast cancer [18–21]. To date, there have been no reports on the alteration of miR-124 expression in papillary thyroid carcinoma. Nevertheless, Nikiforova et al. reported that the expression of miR-124a is significantly up-regulated in medullary thyroid carcinoma tissue [22]. We found that miR-124-3p expression was markedly up-regulated in PTC patients. Although the results of both our and Nikiforova's studies showed a contrasting trend of miR-124 in thyroid cancer compared to the studies in other types of cancer, we proposed that miR-124 might play a crucial role as an oncogene promoter in thyroid cancer. miRNA function in cancer is known to be context-dependent [23, 24]. A given miRNA involved in tumorigenesis can target two groups of genes with distinct functions, depending on the cell type or environment. It can act as an oncogene promoter in some types of tissues and a tumor suppressor in other types, and it also can act as both an oncogene and a tumor suppressor in the same tissue [25]. This ability of miRNAs in the same tissue might be important for normal development and cell differentiation. The underlying mechanisms of the contextual and opposite effects have not been fully elucidated but may be due to the relative abundance of a target mRNA, alternative polyadenylation sites resulting in the loss or gain of miRNA transcript binding sites, or mutation of miRNA binding sites [26].

Dysregulation of miR-9 expression has been reported in many types of cancer, which indicated that miR-9 is involved in tumor formation or progression. Accumulating studies demonstrated that miR-9 had opposite effects on tumorigenesis in different types of cancer cells by regulating different target genes. MiR-9 expression was up-regulated in bladder transitional cell carcinoma, mixed lineage leukemia-rearranged acute myeloid leukemia, hepatocellular carcinoma, primary brain tumors, CDX2-negative gastric cancer cells and breast cancer [27–34]. Murray et al. found that circulating miR-9-3p was over-expressed in MYCN-amplified high-risk neuroblastoma [35]. In contrast, miR-9 was down-regulated in several other types of cancers, such as human ovarian tumor cells [36, 37]. To date, there have been very few studies about the effects of miR-9 on thyroid cancer. Wojtas et al. analyzed the miRNA expression in follicular thyroid carcinomas (FTCs), and found that miR-9-3p showed an about 3.5 fold down-regulated expression in FTCs compared to the normal thyroid tissue from the opposite lobe [38]. He et al. analyzed the expression of miRNAs in PTC tumor tissues. The expression of miR-9–3 in PTC tissues showed a 0.7-fold of that in the normal thyroid tissue adjacent to PTC tumors (local false discovery rates: 7.3%) [39]. Sondermann et al. found that miR-9 was a significant and independent prognostic factor for recurrence in PTC patients [40]. In the present study, we found that the expression of circulating miR-9-3p was significantly up-regulated in PTC patients compared with that in patients with benign nodules and healthy controls. ROC analysis revealed that circulating miR-9-3p had a high diagnostic value for the differentiation of thyroid nodules.

MiR-196b is located in a highly evolutionarily conserved region between the HOXA9 and HOXA 10 genes, at chromosome band 7p15.2 in humans [41]. Chen et al. reported that miR-196b directly targeted both the HOXA9/MEISI oncogenes and FAS tumor suppressor in MLL-rearranged leukemia, and the oncogenic role of miR-196b prevails over its tumor-suppressor role [42]. Thus, the overexpression of miR-196b is associated with aggressive leukemic phenotypes. Up-regulation of miR-196b was also observed in colon cancer and oral cancer [43, 44]. In other words, in some other types of cancer, such as acute lymphoblastic leukemia, chronic myeloid leukemia and breast cancer, the repression of oncogenic targets by miR-196b is more important than its repression of tumor-suppressor targets [45–48]. Down-regulation of miR-196b expression promoted tumorigenesis. There are no reports on the dysregulation of miR-196b expression in thyroid diseases to date. In the present study, we found that the expression of miRNA-196b-5p was down-regulated in PTC patients and up-regulated in patients with benign thyroid nodules by an initial screening with a miRCURY LNA Array. Although further validation by qRT-PCR did not support the down-regulation in PTC patients, the expression of miR-196b-5p was confirmed to be up-regulated in patients with benign nodules compared with that of both healthy controls and PTC patients. ROC analysis also revealed that miR-196b-5p had a good diagnostic value to distinguish benign nodules from PTC.

This study has several limitations: 1) the sample size was small. The findings of this study only provided a clue for further research. 2) The study population only included participants in an eastern coastal area of China. We did not assess circulating miRNAs in different areas and elucidate the effects of different regions on the dysregulation of miRNAs in PTC patients. 3) We did not explore the exact functions of these miRNAs. Further studies with a larger population from different representative areas are needed to evaluate the diagnostic value of these circulating miRNAs for the differentiation of PTC patients from benign ones and to determine whether there is different dysregulation of miRNAs in different regions.

## MATERIALS AND METHODS

### Study population

The study patients with thyroid nodules were derived from a consecutive series of patients with thyroid nodules who underwent thyroidectomy in the Affiliated Yantai Yuhuangding Hospital of Qingdao University, Yantai, China. The final diagnosis was based on a pathological examination. Patients with PTC or benign thyroid nodules were enrolled (*n* = 50 for each group). The healthy controls were enrolled after a neck ultrasound screening and measurements of thyroid hormone (*n* = 50). Exclusion criteria included any other benign or malignant tumors, blood-related diseases, and dysfunction of major organs, such as liver, kidney, lung and heart. The study protocol was approved by the local ethics committee, and written informed consent was obtained from all participants.

### Sample preparations

To investigate the dysregulated expression of circulating miRNAs in PTC patients, peripheral venous blood samples were collected from all participants after enrollment. Plasma was obtained after centrifugation at 4°C and stored at –80°C for further measurements. To determine the expression of the dysregulated circulating miRNAs in thyroid tissue, thyroid tissue samples, including PTC, normal thyroid tissues adjacent to carcinoma and benign thyroid nodule tissues (*n* = 27 for each group), were collected during thyroid surgery and stored at –80°C. To elucidate the alteration of miRNA expression after thyroidectomy, we followed up PTC patients who received a thyroidectomy for 4 weeks (*n* = 24) and collected their blood samples for further qRT-PCR analysis.

### RNA extraction

Total RNA was isolated from blood and thyroid tissues using TRI reagent BD (TB-126, MRCgene) and TRIzol Reagent (Invitrogen), respectively, and purified with an RNeasy mini kit (QIAGEN) according to the manufacturer's instructions. RNA quality and quantity was measured using a Nanodrop spectrophotometer (ND-1000, Nanodrop Technologies), and RNA integrity was determined by gel electrophoresis.

### Plasma miRNA profiling

Three plasma samples were randomly selected from each group to screen the expression of miRNAs. The differential miRNA expression profiling analysis was performed using a miRCURY LNA Array (v.18.0, Exiqon, Denmark) according to the manufacturer's instruction. miRNA labeling and hybridization was performed with 1 μg total RNA of each sample as inputs using a miRCURY Array Power Labeling kit (Exiqon, Denmark) according to the manufacturer's protocol. The slides were washed and scanned using an Axon GenePix 4000B microarray scanner. GenePix Pro V6.0 was used to assess the raw intensity of the images. The intensity of the green signal was calculated after background subtraction, and four replicate spots of each probe on the same slide were averaged. Normalized data were obtained using median normalization, with normalized data = (Foreground-Background)/median. The median is the 50 percent quantile of microRNA intensity, which was larger than 30 in all samples after background correction. After normalization, the statistical significance of differentially expressed miRNA was analyzed by one-way ANOVAs. The plasma miRNA profiling and data analysis were performed at KangChen Bio-tech (Shanghai, China).

### Real-time quantitative RT-PCR of selected miRNAs

MiRNAs shown to have significantly different expression by the miRCURY LNA Array were further validated by real-time quantitative reverse transcription polymerase chain reaction (qRT-PCR) in plasma samples from all participants in each group. The qRT-PCR analysis was also performed with thyroid tissue samples and plasma samples of PTC patients 4 weeks after thyroidectomy to observe the expression of the dysregulated circulating miRNAs in thyroid tissues and the alteration in the plasma after thyroidectomy.

Reverse transcription was performed in a 20 μl reaction system containing 1.5 μg of total RNA, 1.2 μl miRNA-RT primers (1 μM), 10 nmol dNTP Mix, and 0.2 μl MMLV reverse transcriptase (GenePharma). The qRT-PCR analysis of miRNAs was performed using SYBR Green microRNA assays according to the manufacturer's protocol. Primers (5′-3′) used for the detection of the selected miRNAs are shown in Table 5. The expression of miRNAs relative to miR-16 was determined using ΔΔCt-based fold-change calculations.

**Table 5:** Primers of miRNAs for qRT-PCR

Gene	Forward Primer	Reverse Primer
**miR-124-3p**	GCCGCTAAGGCACGCG	TATGGTTGTTCACGACTCCTTCAC
**miR-9-3p**	GGCGCGGAAATAAAGCTAGATA	TATGGTTGTTCACGACTCCTTCAC
**miR-4701**	GCGATGGGTGATGGGTG	TATGGTTTTGACGACTGTGTGAT
**miR-196b-5p**	GCACCAGCGTAGGTAGTTTCC	TATGCTTGTTCTCGTCTCTGTGTC
**miR-16**	AAGCACCTAGCAGCACGTAAATA	TATGGTTTTGACGACTGTGTGAT

### Statistical analysis

Quantitative values are expressed as the mean ± standard error of the mean (SEM) and were analyzed by an unpaired *t-test* or one-way ANOVA as appropriate. Post hoc comparisons were carried out with least significant difference test when equal variances were assumed or with Dunnett's test when equal variances were not assumed. Qualitative data were analyzed by chi-square tests. Receiver operating characteristic curve analysis (ROC) was performed to identify the sensitivity and specificity of a single miRNA to differentiate PTC patients from patients with benign nodules or healthy controls. SPSS 16.0 (SPSS Inc., Chicago, IL, USA) was used for statistical analysis, and *P* < 0.05 was considered significant.

## CONCLUSIONS

In conclusion, the present study revealed that the expression of circulating miR-124-3p and miR-9-3p was substantially up-regulated in PTC patients, while miR-196b-5p was up-regulated in patients with benign nodules in an eastern coastal area of China. ROC analysis indicated that miR-124-3p, miR-9-3p and miR-196b-5p had good diagnostic values to differentiate PTC patients from patients with benign nodules. Our findings provide support for the development of an easy, minimally invasive diagnostic tool for the preoperative differentiation of thyroid nodules.
